# Spontaneous spinal epidural hematoma masquerading as atypical abdominal pain in a child: A case report

**DOI:** 10.1097/MD.0000000000021762

**Published:** 2020-08-14

**Authors:** Xueliang Cheng, Yang Qu, Rongpeng Dong, Lili Yang, Mingyang Kang, Jianwu Zhao

**Affiliations:** The 2nd Hospital of Jilin University, Changchun, Jilin, People's Republic of China.

**Keywords:** abdominal pain, case report, intraspinal hemorrhage, neurological dysfunction, pediatrics

## Abstract

**Introduction:**

There have been few case reports of abdominal pain as a symptom of spontaneous intraspinal hemorrhage. We herein describe a case involving a girl with paraplegia caused by spontaneous epidural hemorrhage in the thoracic spinal canal, characterized by abdominal pain.

**Patient concerns:**

An 8-year-old girl with sudden abdominal pain and back pain was misdiagnosed as having an abdominal disease until she had the symptom of paralysis.

**Diagnoses:**

The patient was diagnosed with spontaneous intraspinal hemorrhage masquerading as atypical abdominal pain.

**Interventions:**

When the patient developed symptoms of lower extremity paralysis, thoracic magnetic resonance imaging was performed and epidural hemorrhage was found in the thoracic spinal canal. Surgical treatment was performed after the diagnosis was confirmed.

**Outcomes:**

The patient could almost walk normally after 3 months. One year after surgery, the Frankel grade of spinal cord function was grade D. We continued to follow-up this patient.

**Conclusion:**

The symptoms caused by intraspinal hemorrhage are mainly back pain with or without neurological dysfunction. However, sometimes atypical symptoms, such as abdominal and chest pain, can be identified in clinical settings. Emergency surgery is recommended as the treatment of choice for intraspinal hemorrhage with neurological dysfunction.

## 1 Introduction

Spontaneous intraspinal hemorrhageis defined as intraspinal hemorrhage without definite trauma or iatrogenic injury. The incidence of the disease is very low (1/100,000 persons),^[1]^ accounting for 0.3% to 0.9% of all spinal cord injuries.^[2]^ The disease has characteristics of acute onset, severe symptoms, and high disability rate. Spontaneous intraspinal hemorrhage mainly occurs in adults older than 40 years.^[3,4]^ We describe a case involving a girl with paraplegia caused by spontaneous epidural hemorrhage in the thoracic spinal canal, characterized by abdominal pain.

## 2 Case report

The patient's parents provided informed consent, and the study design was exempted from the requirement of obtaining approval from the ethics review board. The medical records and imaging study results were subsequently extracted.

The patient was an 8-year-old girl, who complained of abdominal pain and paralysis of the 2 lower extremities for 5 days. She had no history of hemorrhagic disease and no family history of genetic diseases. Her parents said that she had sudden back pain without any obvious causes 5 days before, followed by abdominal pain, and a relief from the back pain 2 hours later. The girl was treated for abdominal pain in a local hospital. However, the symptoms were not relieved, and her condition progressively worsened, with gradual walking difficulties.

**Figure 1:**
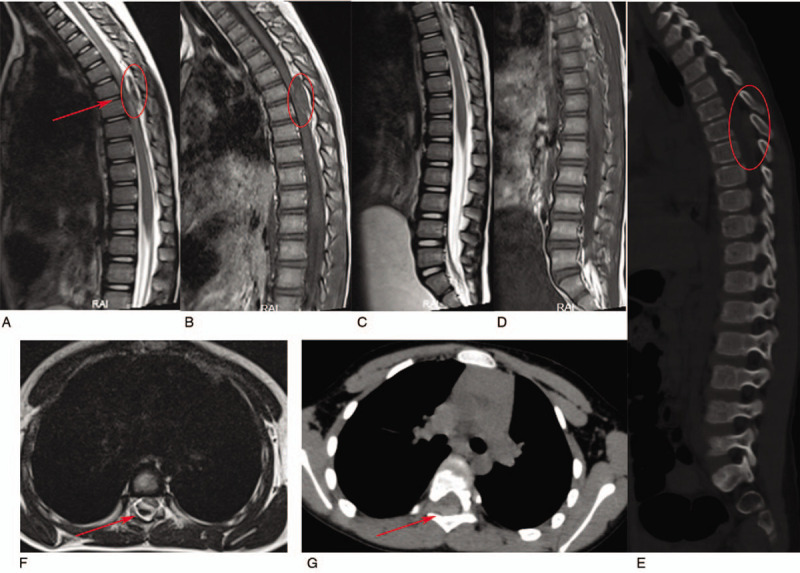
Preoperative images. The magnetic resonance imaging and computed tomography showed an abnormal epidural mass (28 × 9 mm) at the level of T6-7 (red circle and red arrow).

Five days after disease onset, she was transferred to our hospital. Physical examination showed abdominal tenderness and paralysis below the level of both subcostal ribs. The muscle strength of the upper limbs was decreased, and that of both lower limbs was grade 0. Both lower extremity tendon reflexes were absent, the Babinski and lateral Chaddock signs were positive bilaterally, and the Frankel grade of spinal cord function was A. Thoracic magnetic resonance imaging (MRI) showed a slightly short T1 and T2 signal epidural mass (28 × 9 mm) at the level of the T6–7 vertebral body. The adjacent spinal cord was compressed, and a long T2 signal was noted in the spinal cord. The bladder volume had increased significantly, and the uterus was not visible (Fig. 1). Thoracic computed tomography (CT) showed abnormal soft tissue located at the level of the thoracic 6–7 vertebral body. The CT value of the mass was 68 HU with a maximum cross section of 14 × 7 mm, and the adjacent dural sac was compressed (Fig. 1). Abdominal CT showed no obvious abnormalities. The initial diagnosis was an intraspinal occupying lesion. Results of the coagulation function test were normal. An emergency surgery was then performed.

**Figure 2:**
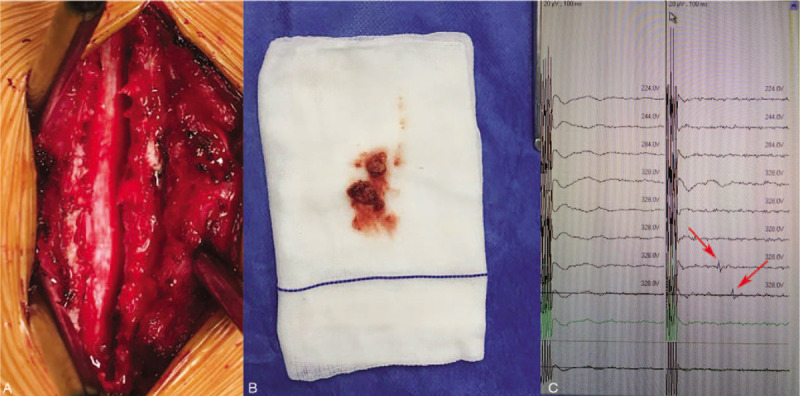
Perioperative images. (A) Spinal canal morphology after hematoma removed. (B) Blood clot. (C) The waveform of motor evoked potential was detected by intraoperative neurophysiological monitoring at the end of operation (red arrow).

We observed varicose blood vessels and the mass located at the dorsal side of the epidural spine (Fig. 2). The intraoperative neurophysiological examination results did not change significantly when the lamina was removed, and the hematoma resolved. At the end of the operation, the neurophysiological examination showed a conductive electrical signal-waveform of motor evoked potential (Fig. 2). The histopathological examination of the tissue biopsy confirmed that this mass was a hematoma.

**Figure 3:**
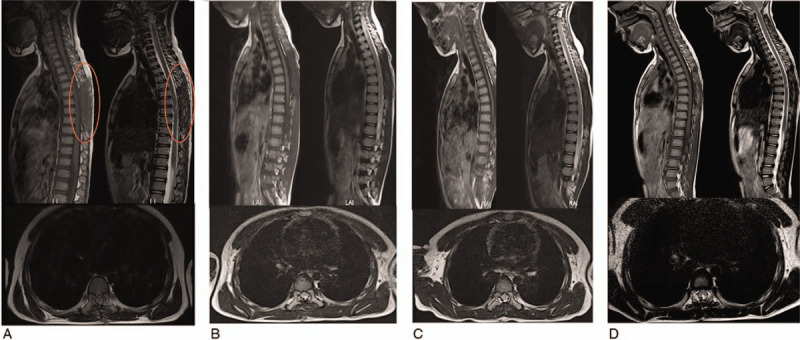
The magnetic resonance imagings at different timepoints after operation. (A) 1 m, (B) 3 m, (C) 6 m, (D) 12 m. The area shown in the red circle is the operation area.

**Figure 4:**
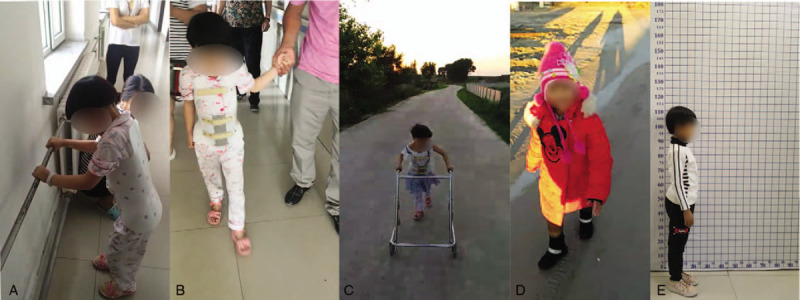
Function recovery images at different timepoints after operation. (A) 0.5 m, (B) 2 m, (C) 3 m, (D) 6 m, (E) 12 m.

Postoperative treatment included neurotrophic treatment, antibiotics, fluids for dehydration, anti-inflammatory medication, and rehabilitation training. Postoperative abdominal pain was significantly relieved. Five days postoperatively, the patient felt pain in her left lower extremity, and abdominal pain reappeared. It was thought that the abdominal pain was caused by appendicitis or urinary tract infection; thus, she was transferred to pediatrics for further treatment. MRI was regularly conducted during postoperative follow-ups, as shown in Figure 3, and the patient's functional recovery processes are shown in Figure 4. On the 10^th^ day after surgery, muscle contraction occurred in the left lower extremity (muscle strength grade 1); 12 days after surgery, muscle contraction occurred in the right leg (muscle strength grade 1), and she had hyperalgesia in the lower limb with a muscle strength in both lower limbs of grades 2–3. Bladder function partially recovered. One month after surgery, the muscle strength of both lower limbs was restored to grade 4. One and a half months post-operation, she could walk with assistance. Three months after surgery, she could walk without any help. Bladder function was normal. We instructed the patient to continue to perform functional rehabilitation exercises and be careful to prevent spinal deformity. A year later, the girl could walk around without assistance, the muscle strength of both lower extremities was normal. The sensory sensitivity of the skin below the rib arch was slightly worse than that of the normal skin. The Babinski and Chaddock signs were positive bilaterally, and the Frankel grade was D.

## 3 Discussion

Here, we reported the case of an 8-year-old girl with spinal epidural hematoma masquerading as atypical abdominal pain who was successfully treated by surgical interventions. Because of the atypical symptoms, the patient was misdiagnosed. Since the patient was younger, the spinal cord plasticity was stronger, and the clinical outcome was better after emergency operation.

The main causes of spontaneous intraspinal hemorrhage include vascular malformation, blood system diseases, anticoagulant therapy, pregnancy, and so on. Emotional agitation, such as hysterical crying, is a predisposing factor for the disease, but those mainly affected are patients younger than 2 years old.^[5]^ This patient had no familial hypertension or obvious trauma, received no anticoagulant medication, and had no vascular disease. Thus, vascular malformation is considered the main cause according to the intraoperative findings. Vascular malformation is the second most common cause in patients younger than 15 years.^[6]^ The intraspinal venous plexus, which is located on the dorsal side of the spinal cord, is relatively weak and sensitive to pressure fluctuations.^[7]^ Additionally, the veins of the abdomen and chest are connected to the intervertebral vein.^[8]^ Kreppel et al^[6]^ believes that a specific location of intervertebral vascular infarction and abnormal high blood vessel pressure lead to spontaneous intraspinal hemorrhage or bleeding. Therefore, in our case, we think the patient was in a squatting position and the spine was in a state of flexion, which reduced the volume of the corresponding spinal canal; consequently, the pressure in the spinal canal and abdominal pressure increased simultaneously, leading to rupture of the blood vessels in the spinal canal.

In this case, the reasons for misdiagnosis were as follows: first, abdominal pain is a rare and atypical symptom of the disease. Lee and Choi^[9]^ and Tsen et al^[10]^ described cases wherein intraspinal hemorrhage with chest pain as the main complaint was misdiagnosed as myocardial infarction. The mechanism is that the pain was caused by the hematoma irritating the nerve root or the anterior bundle of the spinal cord. Secondly, intraspinal hemorrhage always leads to paralysis.^[6]^ Similar diseases include Guillain-Barré syndrome and acute myelitis. Kondo et al reported a case of intraspinal hemorrhage that was misdiagnosed as Guillain-Barré syndrome.^[11]^ Thirdly, this patient was very young, so the possibility of intraspinal hemorrhage was very low.

The mainstream view on the treatment of intraspinal hemorrhage is that surgical treatment should be actively performed after the diagnosis is clearly established,^[12,13]^ and the operation should be performed within 72 hours after the onset of bleeding.^[6]^ Although there are reports of conservative treatment,^[2,14]^ incomplete hematoma absorption may occur secondary to spinal canal stenosis or affect spinal cord recovery. Postoperative prognosis is related to the progress of the patient's disease course, degree of spinal cord compression, timing of surgery, and patient's age.^[15,16]^

## 4 Conclusions

The symptoms caused by intraspinal hemorrhage are mainly acute back pain with or without neurological dysfunction. Sometimes atypical symptoms, such as abdominal and chest pains, should be identified in clinical settings.

## Acknowledgments

The authors thank Editage for -polishing the paper.

## Author contributions

**Conceptualization:** Xueliang Cheng

**Data curation:** Yang Qu, Rongpeng Dong.

**Formal analysis:** Xueliang Cheng, Lili Yang.

**Methodology:** Mingyang Kang.

**Supervision:** Jianwu Zhao.

**Writing – original draft:** Xueliang Cheng, Yang Qu, Rongpeng Dong, Lili Yang

**Writing – review & editing:** Jianwu Zhao
